# Different transcriptional response between susceptible and resistant common carp (*Cyprinus carpio*) fish hints on the mechanism of CyHV-3 disease resistance

**DOI:** 10.1186/s12864-019-6391-9

**Published:** 2019-12-26

**Authors:** Roni Tadmor-Levi, Adi Doron-Faigenboim, Evgeniya Marcos-Hadad, Jules Petit, Gideon Hulata, Maria Forlenza, Geert F. Wiegertjes, Lior David

**Affiliations:** 1 Department of Animal Sciences, RH Smith Faculty of Agriculture, Food and Environment, The Hebrew University of Jerusalem, Rehovot, Israel; 2 National Natural History Collections and Department of Ecology, Evolution and Behavior, The Hebrew University of Jerusalem, Jerusalem, Israel; 3 Agricultural Research Organization, Volcani Center, Rishon LeZion, Israel; 4 Cell Biology and Immunology Group, Wageningen University & Research, Wageningen, Netherlands; 5 Aquaculture and Fisheries Group, Wageningen University & Research, Wageningen, Netherlands

**Keywords:** Fish disease, Aquaculture, Koi herpes virus (KHV), Disease resistance, Wild-type strain, RNA sequencing, Chemokines, Neutrophils, Immuno-genetics

## Abstract

**Background:**

Infectious disease outbreaks form major setbacks to aquaculture production and to further development of this important sector. Cyprinid herpes virus-3 (CyHV-3) is a dsDNA virus widely hampering production of common carp (*Cyprinus carpio*), one of the most farmed fish species worldwide. Genetically disease resistant strains are highly sought after as a sustainable solution to this problem. To study the genetic basis and cellular pathways underlying disease resistance, RNA-Seq was used to characterize transcriptional responses of susceptible and resistant fish at day 4 after CyHV-3 infection.

**Results:**

In susceptible fish, over four times more differentially expressed genes were up-regulated between day 0 and 4 compared to resistant fish. Susceptible and resistant fish responded distinctively to infection as only 55 (9%) of the up-regulated genes were shared by these two fish types. Susceptible fish elicited a typical anti-viral response, involving interferon and interferon responsive genes, earlier than resistant fish did. Furthermore, chemokine profiles indicated that the two fish types elicited different cellular immunity responses. A comparative phylogenetic approach assisted in chemokine copies annotation pointing to different orthologous copies common to bony-fishes and even carp-specific paralogs that were differentially regulated and contributed to the different response of these two fish types. Susceptible fish up-regulated more *ccl19* chemokines, which attract T-cells and macrophages, the anti-viral role of which is established, whereas resistant fish up-regulated more *cxcl8/il8* chemokines, which attract neutrophils, the antiviral role of which is unfamiliar.

**Conclusions:**

Taken together, by pointing out transcriptional differences between susceptible and resistant fish in response to CyHV-3 infection, this study unraveled possible genes and pathways that take part in disease resistance mechanisms in fish and thus, enhances our understanding of fish immunogenetics and supports the development of sustainable and safe aquaculture.

## Background

Securing a steady supply of healthy and nutritious foods for the growing human population is one of the main challenges of today and aquaculture takes a growing share in addressing this challenge [1]. Major impediments in sustainable production and further growth of the food-fish sector are caused by a whole range of infectious diseases [2], because measures for their prevention and control under aquaculture conditions are very limited. Effective ways to alleviate the problem and improve sustainable production include vaccines development [3–6] and breeding of disease resistant strains [7–10].

Common carp (*Cyprinus carpio*), henceforth referred to as ‘carp’, is one of the five most produced fish species worldwide and an important food source in many heavily populated low-income countries. A major threat to aquaculture of both food strains and ornamental Koi varieties is a disease caused by the cyprinid herpes virus type 3 (CyHV-3), also called Koi herpes virus (KHV), which belongs to the double-stranded DNA Alloherpesviridae family. The disease is specific to common carp, however, the asymptomatic presence of this virus has been recorded in several other cyprinid species. Outbreaks of CyHV-3 have started in the late 1990s and have been persisting since then, spreading to most environments in which carp is cultured and causing significant losses of up to 100% of the pond fish [11–13]. Such a widespread distribution of outbreaks indicates that carp strains used in aquaculture are generally susceptible to the disease. Several studies noted the susceptibility of cultured strains in contrast to the resistance of a feral strain [14–19]. The virus successfully reproduces in susceptible fish, causing loss of appetite, tissue damage, skin lesions, and finally mortalities. Similarly to susceptible fish, also resistant ones get infected by the virus. However, in contrast to susceptible fish, resistant ones suffer less damage and are able to recover from the disease. Significantly lower viral load was found in resistant fish compared to susceptible fish, suggesting that resistant fish have a mechanism to restrict virus replication in their tissues [18].

The little information existing so far on mechanisms conferring disease resistance in fish points to lower viral entry, as in the case of infectious pancreatic necrosis virus (IPNV) in Atlantic salmon (*Salmo salar*) [20], and/or to improved immune response to infection [7, 8, 21–23]. As for other vertebrates, also in fish, specific immune responses and immune mechanisms will be required to mount a protective response against infectious pathogens. However, the protective response in fish might have a more complex genetic basis since the bony-fishes specific whole genome duplication further contributed to the degree of complexity of the immune repertoire, especially when considering the expansion in the number of interleukins (ILs), Toll-like receptors (TLRs) and chemokines [24–26]. Common carp, having gone through one additional recent whole genome duplication [27], potentially has an even more diverse immune gene repertoire than diploid fish species [28]. The common carp genome was sequenced, but the current assemblies are still discontinuous and comprised of many scaffolds [29–31]. The current carp transcriptome [30] includes about 50,000 protein-coding transcripts annotated based primarily on sequence homology to the model zebrafish (ZF) (*Danio rerio*).

A few studies have uncovered some genetic differences between carp fish that survived or died from CyHV-3 infection. Using candidate gene approaches, differences were found between survivor and dead fish in allelic frequencies for *cyca-DAB1-like* [32] and *il10* [33] genes, which represent both the adaptive and innate immune responses, respectively. Genotyping-by-Sequencing was used for quantitative trait loci (QTL) analyses yielding a few QTLs with relatively mild effects on survival [34, 35]. However, to date most of the genetic variation affecting this trait remains unknown.

Therefore, in this study, RNA sequencing (RNA-Seq) was applied to compare the transcriptomic response to CyHV-3 infection between fish from susceptible and resistant families with, the goal to identify possible pathways underlying resistance in carp. While susceptible fish elicited a typical anti-viral response, resistant fish elicited cellular immunity involving neutrophils that might be a part of a novel resistance mechanism. In addition, some of our other results resemble findings in diseases of other species and thus, suggest commonalities that together enhance our understanding of viral resistance mechanisms in fish.

## Results

### Transcriptome differences between susceptible and resistant fish

**Fig. 1:**
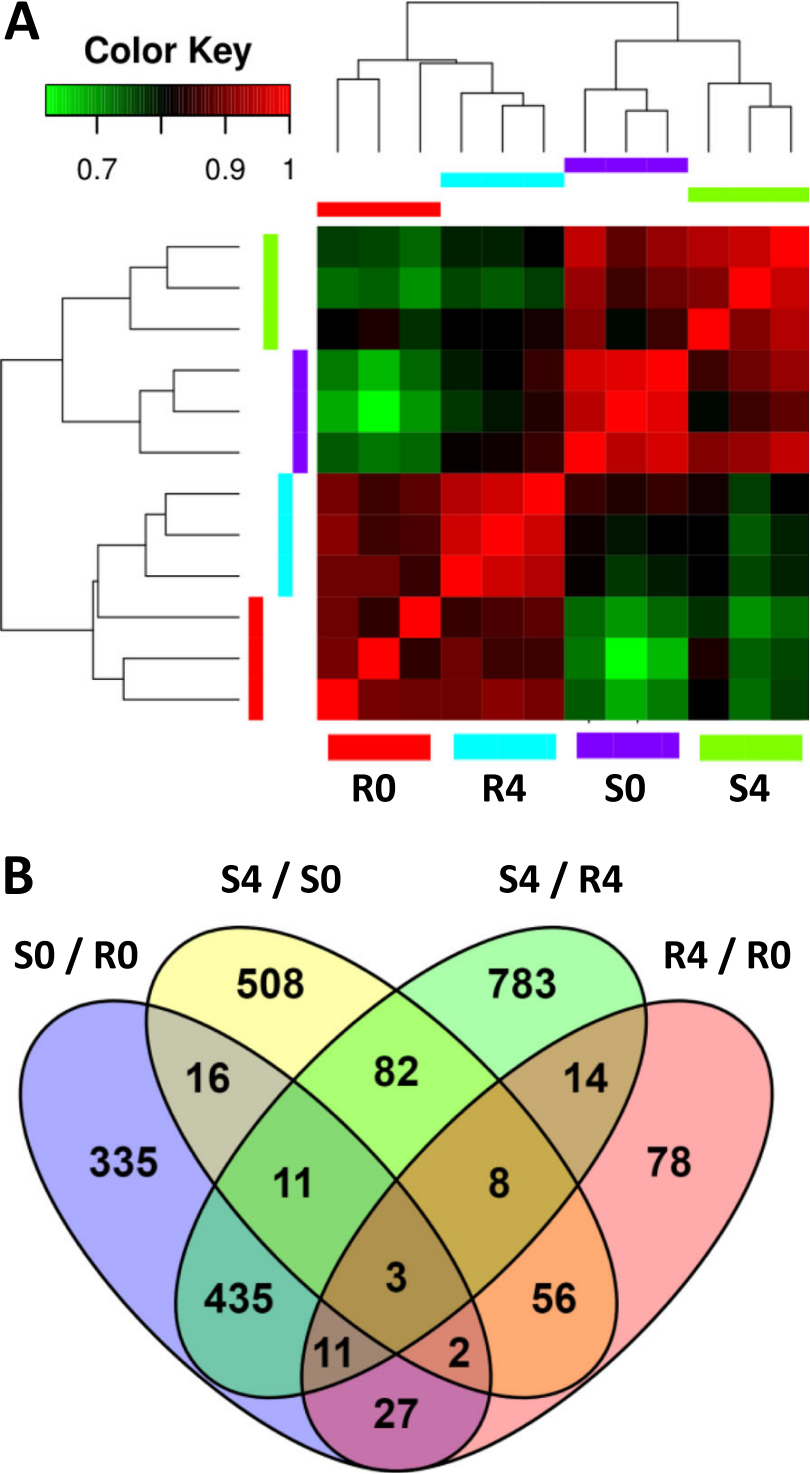
Similarity and overlaps in differentially expressed genes among experimental treatments. Four treatments were analyzed each in three replicates: Resistant at day 0 (R0) and day 4 (R4) and Susceptible at day 0 (S0) and day 4 (S4). **a** Similarity among replicates and treatments is shown by a heat map of correlation coefficients between normalized read counts of differentially expressed genes (DEGs). **b** Number of DEGs identified in each of four pairwise comparisons (S0/R0, S4/S0, S4/R4 and R4/R0) and the overlaps among these DEG lists

RNA sequencing was conducted on samples of spleen RNA pooled from three fish each. Such pools were constructed from three susceptible (S) and three resistant (R) families at two timepoints: pre-infection (day 0), and 4 days post infection (day 4). In a clustering analysis, based on normalized read counts of differentially expressed genes (DEGs), the three replicates of susceptible families were highly correlated among themselves and so were the three replicates of resistant families, thus, attesting to the high reproducibility of the data (Fig. 1a). Furthermore, higher correlations (red color) were found within fish types (susceptible or resistant) than within timepoints (pre- or post-infection), demonstrating the many transcriptional differences between these fish types, even without the infection (Fig. 1a). In the four comparisons between pairs of treatments (R0/S0, S4/R4, S4/S0 and R4/S4), a total of 2369 DEGs were found. Relatively little overlap of DEGs between the four comparisons indicated that transcriptomes of the four treatments were considerably distinct (Fig. 1a). For instance, only three genes were shared among all four comparisons, while 1704 (72%) were comparison-specific. The largest overlap (435 DEGs) was found between S0/R0 and S4/R4, which represented fish-type differences regardless of the disease infection. The overlap between fish-types in response to infection (S4/S0 ∩ R4/R0) included only 69 DEGs.

**Fig. 2:**
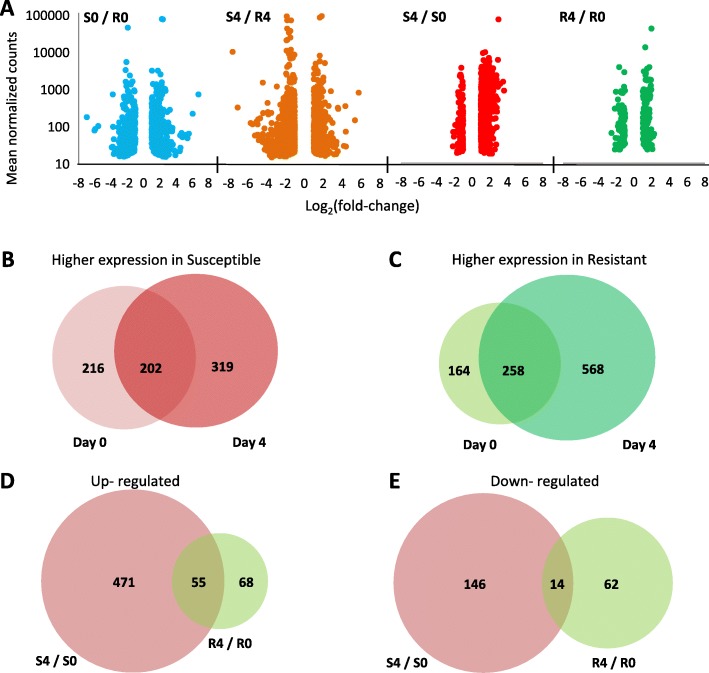
Volcano plots of DEGs and overlaps in DEGs in response to infection. **a** Volcano plots showing the distribution of normalized read counts as a function of Log_2_(fold-change) for DEGs in each of the four comparisons. Negative and positive Log_2_(fold-change) describe down-regulated and up-regulated genes, respectively, relative to the reference treatment, which is always the denominator of the title (e.g for S0/R0, R0 is the reference). **b** and **c** Overlap in number of genes more highly expressed at days 0 and 4 (**b**) in susceptible compared to resistant fish and **c** in resistant compared to susceptible fish. **d** and **e** Overlap in number of genes between the response to infection of resistant (R4/R0) and susceptible (S4/S0) fish divided to (**d**) up-regulated and (**e**) down-regulated DEGs

Each of the four comparisons identified both up- and down-regulated genes relative to a reference treatment (the denominator of each comparison), all with a wide distribution of normalized read counts (Fig. 2a). Some genes (*n* = 737) were more highly expressed in susceptible fish (Fig. 2b), whereas other genes (*n* = 990) in resistant ones (Fig. 2c). Only about ¼ of these genes (202/737 for susceptible and 258/990 for resistant) were shared between days 0 and 4, indicating that the transcriptional differences between fish types changed considerably in response to infection. These changes in expression between days 0 and 4 are reflecting the response to infection, and this response differed considerably between susceptible and resistant fish in two aspects. First, many more genes were differentially expressed in susceptible (*n* = 686) than in resistant (*n* = 199) fish. This broader response of susceptible fish applies to both up- and down-regulated genes (Fig. 2d and e). Second, there was little overlap in the set of DEGs between susceptible and resistant fish. Only 9.3% (*n* = 55) and 6.3% (*n* = 14) of the up- and down-regulated DEGs, respectively, were shared between these fish types (Fig. 2d and e). Therefore, in addition to baseline differences due to their different genetic background, these fish types had elicited a distinctive transcriptional response to CyHV-3 infection.

### GO terms enriched with DEGs in response to infection

**Fig. 3:**
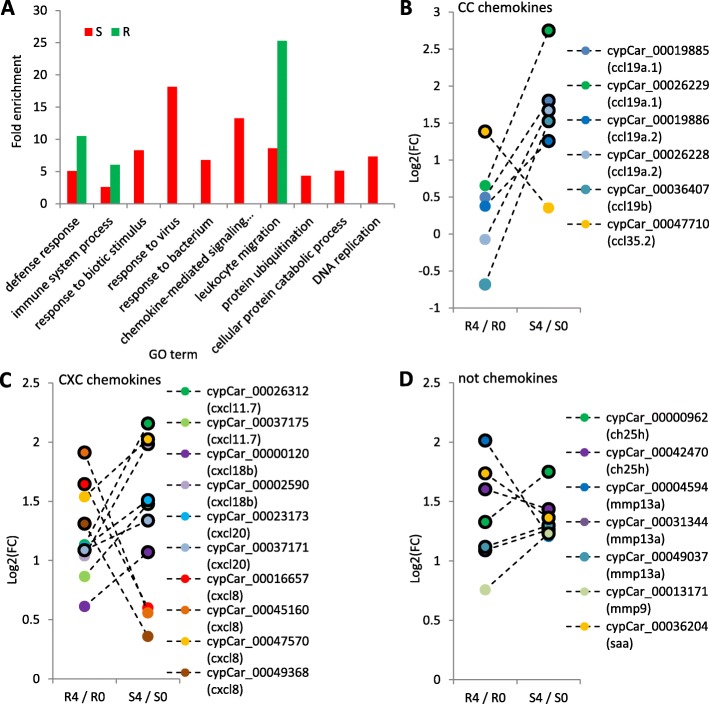
Enriched GO terms and expression levels of DEGs from enriched terms. **a** Fold enrichment of GO terms significantly enriched for up-regulated genes in response to infection (day4/day0) in susceptible (red bars) and resistant (green bars) fish. **b-d** Log_2_(fold-change) between days 4 and 0 of carp genes from the enriched ‘leukocyte migration’ GO term. Genes are marked by different colors and cypCar IDs (unique identifier of a gene module in the common carp genome). Gene name based on ZF annotation is given in parentheses. Different cypCars with the same gene name, represent different carp copies with highest homology to the same ZF gene. Dashed lines connect the differences in expression levels [Log_2_(FC)] of each gene in response to infection in resistant (R4/R0) and susceptible (S4/S0) fish. Genes that were found to be DEGs between days 4 and 0 are marked by black-outlined dots. Note that a value of 0 means no change, a value of 1 means 2-fold change and larger than 1, a higher fold change. Such comparisons were done for different gene families: (**b**) CC chemokines, (**c**) CXC chemokines and (**d**) non-chemokine genes included in this GO term

The response to CyHV-3 infection is represented by the transcriptional differences elicited after infection and thus, gene ontologies (GO) of up-regulated gene lists between days 0 and 4 were studied (Fig. 2d). In the list of 55 genes shared between susceptible and resistant fish, the only significantly enriched GO term was ‘response to stress’ (GO:0006950), which reflects the general response of the fish to the challenge conditions. The lists of 471 and 68 DEGs specific to the response of susceptible and resistant fish, respectively, were both enriched in up-regulated DEGs belonging to immunity-related GO terms (Fig. 3a). For susceptible fish, the most enriched term was ‘response to virus’ (GO:0009615) with 20 DEGs in carp that correspond to nine genes out of 48 included in this term based on the ZF annotation. The majority of these DEGs were interferons (IFNs) and interferon stimulated genes (ISGs). Another highly enriched term for susceptible fish was ‘chemokine-mediated signaling pathway’ (GO:0070098), with 11 DEGs in carp that correspond to seven out of 51 included in this term based on the ZF annotation (see Table 1 for a gene list of susceptible fish DEGs included in these terms). Other less enriched terms, including some not directly related to immune response, were also found for susceptible fish (Fig. 3a).

**Table 1:** Carp genes (cypCars) in the top enriched GO terms in susceptible fish

GO term	cypCar ID	Description (Blast2GO)	Gene name	ZF Ensembl ID
response to virus (GO:0009615)	cypCar_00007337	interferon regulatory factor 7	*irf7*	ENSDARG00000045661
	cypCar_00033848	interferon regulatory factor 3-like isoform X1	*irf7*	ENSDARG00000045661
	cypCar_00023289	interferon-induced with tetratricopeptide repeats 1-like	*ifit8*	ENSDARG00000057173
	cypCar_00009225	interferon-induced with tetratricopeptide repeats 1-like	*ifit8*	ENSDARG00000057173
	cypCar_00036002		*eif2ak2*	ENSDARG00000068729
	cypCar_00046009	dsRNA-activated kinase R	*eif2ak2*	ENSDARG00000068729
	cypCar_00039221	interferon- double-stranded RNA-activated kinase-like	*eif2ak2*	ENSDARG00000068729
	cypCar_00048805	Z-DNA binding kinase	*Pkz*	ENSDARG00000052396
	cypCar_00042977	Z-DNA binding kinase	*Pkz*	ENSDARG00000052396
	cypCar_00042978	Z-DNA binding kinase	*Pkz*	ENSDARG00000052396
	cypCar_00047118	tumor necrosis factor-like	*Tnfa*	ENSDARG00000009511
	cypCar_00000963	interferon-induced with tetratricopeptide repeats 5-like	*ifit10*	ENSDARG00000007467
	cypCar_00024055	radical S-adenosyl methionine domain-containing 2	*rsad2*	ENSDARG00000004952
	cypCar_00043425		*rsad2*	ENSDARG00000004952
	cypCar_00017679	interferon-induced GTP-binding Mx-like	*Mxe*	ENSDARG00000014427
	cypCar_00021056	lymphocyte antigen 86-like	*ly86*	ENSDARG00000090649
	cypCar_00005064	interferon gamma	*ifng1–2*	ENSDARG00000024211
	cypCar_00017692	interferon gamma	*ifng1–2*	ENSDARG00000024211
	cypCar_00000964		*ifit11*	ENSDARG00000090537
	cypCar_00039376	interferon-induced with tetratricopeptide repeats 5-like	*ifit11*	ENSDARG00000090537
chemokine mediated signaling pathway (GO:0070098)	cypCar_00036407	CC motif chemokine 19-like	*PP2A3*	ENSDARG00000039351
	cypCar_00047570	interleukin-8-like	*il8l1*	ENSDARG00000102299
	cypCar_00000120		*cxcl18b*	ENSDARG00000075045
	cypCar_00002590	CXC motif chemokine 11-like	*cxcl18b*	ENSDARG00000075045
	cypCar_00037175	CXC motif chemokine 11-like	*cxcl11.7*	ENSDARG00000093779
	cypCar_00026312	CXC motif chemokine 11-like	*cxcl11.7*	ENSDARG00000093779
	cypCar_00023173	CXC motif chemokine 10-like	*cxcl20*	ENSDARG00000075163
	cypCar_00019885	CC motif chemokine 19-like	*ccl19a.1*	ENSDARG00000058389
	cypCar_00026229		*ccl19a.1*	ENSDARG00000058389
	cypCar_00019886	CC motif chemokine 19-like	*ccl19a.2*	ENSDARG00000035632
	cypCar_00026228	CC motif chemokine 19-like	*ccl19a.2*	ENSDARG00000035632

Much fewer up-regulated DEGs were found in resistant fish, resulting in only three significantly enriched GO terms, all of which were immune response related (Fig. 3a). These three terms were enriched also for susceptible fish, but, since tests were done on non-overlapping gene lists, these common terms were found to be enriched due to different genes, hence also their fold enrichment and test significance were different. For resistant fish, the highest fold enrichment was for ‘leukocyte migration’ (GO:0050900) with five carp genes that correspond to four out of 90 included in this term based on the ZF annotation. For susceptible fish, a lower fold enrichment of this term was found based on twelve carp genes that overlapped with those in the ‘chemokine-mediated signaling pathway’ term (GO:0070098).

Because ‘leukocyte migration’ was enriched in both fish types, we analyzed in more detail all DEGs in this term, including five DEGs specific to resistant, twelve to susceptible, and six shared by both (see Table 2 for a detailed gene list). Sixteen out of these 23 DEGs were chemokine genes, including six CC and ten CXC types. Susceptible and resistant fish were different both in how many chemokines were up-regulated and also in which types. Of the six CC chemokines, the five which were up-regulated in susceptible fish were *ccl19* homologs and the one in resistant fish was a different chemokine, *ccl35* (Fig. 3b). Of the ten CXC chemokines, two homologs of *cxcl11,* two of *cxcl18*, one of *cxcl20* and one of *cxcl8,* also known as interleukin-8 (*il8*), were up-regulated in susceptible fish*,* whereas in resistant fish, three homologs of *cxcl8/il8* were up-regulated in response to infection. In addition, one homolog of *cxcl20* was up-regulated in both fish types (Fig. 3c). Of the seven non-chemokine genes in this GO term, five were up-regulated in both susceptible and resistant fish (Fig. 3d). Altogether, these data provide further details on the general as well as specific immune responses, differentiating susceptible from resistant fish. While susceptible fish mounted a broad response with clear signals of a viral infection, the response of resistant fish was associated with a specific chemokine-mediated, white blood cells migration response.

**Table 2:** Up-regulated DEGs included in the ‘leukocyte migration’ GO term

up-regulated in:	cypCar ID	Description (B2G)	Gene name	ZF Ensembl ID
Susceptible	cypCar_00000120		*cxcl18b*	ENSDARG00000075045
	cypCar_00002590	CXC motif chemokine 11-like	*cxcl18b*	ENSDARG00000075045
	cypCar_00013171	matrix metallo ase-9	*mmp9*	ENSDARG00000042816
	cypCar_00019885	CC motif chemokine 19-like	*ccl19a.1*	ENSDARG00000058389
	cypCar_00019886	CC motif chemokine 19-like	*ccl19a.2*	ENSDARG00000035632
	cypCar_00023173	CXC motif chemokine 10-like	*cxcl20*	ENSDARG00000075163
	cypCar_00026228	CC motif chemokine 19-like	*ccl19a.2*	ENSDARG00000035632
	cypCar_00026229		*ccl19a.1*	ENSDARG00000058389
	cypCar_00026312	CXC motif chemokine 11-like	*cxcl11.7*	ENSDARG00000093779
	cypCar_00036407	CC motif chemokine 19-like	*ccl19b*	ENSDARG00000039351
	cypCar_00037175	CXC motif chemokine 11-like	*cxcl11.7*	ENSDARG00000093779
	cypCar_00047570	interleukin-8-like	*cxcl8*	ENSDARG00000102299
Resistant	cypCar_00004594	collagenase 3-like	*mmp13a*	ENSDARG00000012395
	cypCar_00016657	interleukin 8	*cxcl8*	ENSDARG00000102776
	cypCar_00045160	tumor-induced factor	*cxcl8*	ENSDARG00000102299
	cypCar_00047710	monocyte chemotactic 1B-like	*ccl35.2*	ENSDARG00000070378
	cypCar_00049368	interleukin-8-like	*cxcl8*	ENSDARG00000102299
Both	cypCar_00031344	collagenase 3-like	*mmp13a*	ENSDARG00000012395
	cypCar_00049037		*mmp13a*	ENSDARG00000012395
	cypCar_00037171	CXC motif chemokine 10-like	*cxcl20*	ENSDARG00000075163
	cypCar_00036204	serum amyloid A	*Saa*	ENSDARG00000045999
	cypCar_00042470	cholesterol 25-hydroxylase	*ch25h*	ENSDARG00000045190
	cypCar_00000962	cholesterol 25-hydroxylase	*ch25h*	ENSDARG00000045190

### Expression pattern of duplicated chemokine genes

**Fig. 4:**
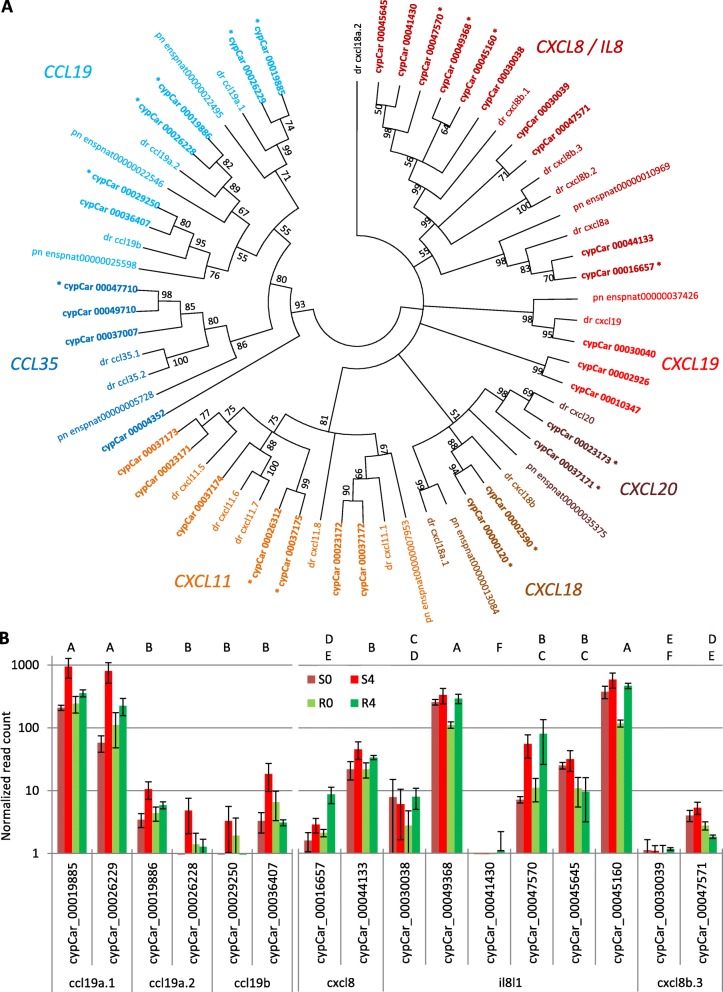
Phylogenetic tree of chemokines and expression levels of their gene copies. **a** A maximum likelihood phylogenetic tree of chemokine genes that at least one of their copies was a DEG in response to infection. The tree includes carp transcripts (cypCar ID), and their homologs from ZF (dr and gene name) and, as an outgroup, homologs from red-bellied piranha (pn and Ensembl ID). Bootstrap values (%) are given at branching points. Different clades of the tree are different chemokines, colored and named after ZF gene family. Note that ZF gene *cxcl18a.2* does not cluster with other *cxcl18* homologs and therefore, might be a wrong annotation. Asterisks adjacent to cypCar IDs mark significantly up-regulated genes in response to infection (in either fish type). **b** Level of gene expression for carp copies of *ccl19* and *cxcl8/il8* gene families. For each homolog of ZF, expression of carp copies are plotted (normalized read count means and standard error bars) for each of the combinations of fish type (R/S) and day (0/4). Carp genes not sharing the same capital letters on top have significantly different expression levels. Note, the evolutionary relationship among gene copies of carp, the expression levels of which shown in (**b**) are derived from (**a**)

Chemokines form a large gene family with several homologs for many of the different family members in genomes of bony-fishes. Since the common carp is a tetraploid species, two paralogous copies are often found in its genome per every ortholog in the genome of its closely related species, the diploid ZF. Since carp genes are annotated mostly after their homologs in ZF, in the case of gene families such as chemokines, separate carp genes (cypCars) are annotated with the same ZF gene name. To better understand the possible contribution of chemokine homologs and of carp-specific paralogs to resistance, the phylogenetic relationships between cypCar chemokines were studied. For all chemokines that one or more of their copies were found to be DEGs, DNA sequences of their transcripts from carp were extracted along with their orthologs in ZF and in red-bellied piranha as an outgroup. Sequences were aligned to construct a phylogenetic tree (Fig. 4a). Based on how the evolutionary relationships among cypCars were resolved by the phylogenetic tree, differences in expression levels between carp copies within a gene clade were studied at three levels.

First, the proportion of DEGs out of the total gene copies was examined. Interestingly, different chemokine types had different proportions of their homologs changing expression in response to infection. For instance, 5/6 *ccl19* homologs, 2/2 *cxcl18* and 2/2 *cxcl20* were DEGs. Conversely, only 1/4 *ccl35* and 2/7 *cxcl11* homologs were DEGs. Among the largest family, *cxcl8/il8*, 4/10 homologs were DEGs (Fig. 4a). Thus, different chemokine families showed different levels of expression divergence among their members.

Secondly, the specific expression differences of all *ccl19* and *cxcl8/il8* homologs in carp were tested using their normalized read counts. Significant differences in expression levels between genes within the *ccl19* and *cxcl8/il8* gene clades were found (all pairs Tukey-Kramer HSD, *P* < 0.05, Fig. 4b). Among *ccl19* homologs, four had a similar and very low expression level compared to two other homologs with significantly higher expression. Among *cxcl8/il8* homologs, expression levels were diverse, ranging from nearly zero to almost 1000 normalized read count. Thus, different chemokines diversified to a different extent in the expression of their homologs.

Thirdly, specific expression patterns of paralogs, derived from the carp-specific whole genome duplication, were analyzed. For most genes, two different carp copies shared a tree branch with a single ZF gene, supporting that these are likely carp-specific paralogs. In the ZF genome, three *ccl19* homologs are annotated: *ccl19a.1* and *ccl19a.2* are tandem duplicates on chr. 5 and *ccl19b* is on chr. 10. For each of these genes, two carp paralogs were found (Fig. 4a). Insignificant differences in expression between carp paralogs were found for all three *ccl19* homologs (all pairs Tukey-Kramer HSD, *P* > 0.05; Fig. 4b). For *cxcl8/il8* there were four annotated ZF genes: *cxcl8a* on chr. 1, *cxcl8b.1* and *cxcl8b.3* are tandem duplicates on chr. 7 and *cxcl8b.2* is unmapped according to the latest version of the ZFIN.org database. In carp, this gene family had further diversified to include ten copies. This family expansion was probably a result of the carp-specific whole genome duplication (as might be suggested by the 2 in carp:1 in ZF ratio observed for *cxcl8a)*, and of specific duplications and losses of specific copies (as for the 1:6 ratio for *cxcl8b.1* or 1:1 ratio for *cxcl8b.2* and *cxcl8b.3)*. Consequently, it was hard to define what genes are paralogs derived from the whole genome duplication and thus, only two *cxcl8/il8* copies of carp that had the 2:1 ratio were analyzed as such paralogs. The expression levels between cypCar_00016657 and cypCar_00044133 differed significantly (all pairs Tukey-Kramer HSD, *P* > 0.05; Fig. 4b) and only cypCar_00016657 was a DEG between susceptible and resistant fish. Thus, unlike for *ccl19*, some level of functional divergence was observed between *cxcl8/il8* carp paralogs, which potentially contributed to resistance.

In summary, using a phylogentic approach to guide the expression analysis, differences in response to infection were revealed among homologs of certain chemokines and even some functional divergence between carp-specific paralogs. Some of these transcriptional differences in homologs and paralogs were DEGs between susceptible and resistant fish.

### DEGs within previously identified QTLs

**Fig. 5:**
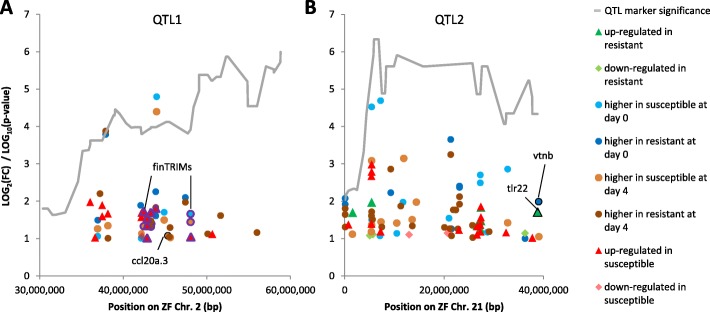
Location and expression of DEGs within previously identified CyHV-3 survival QTLs. Log_2_(fold-change) for DEGs is plotted against their orthologous location in the ZF genome. Plots are shown for (**a**) QTL1 (ZF chromosome 2) and (**b**) QTL 2 (ZF chromosome 21). DEGs location is displayed in the context of the QTL shape (grey line) as deduced from the significance level [Log_10_(*p*-value)] of the association test between markers and survival. Marker position in the ZF genome and their test significance were adapted from [35]. DEG symbols were labeled by shape and color according to the comparison in which they were found significant and their direction (up or down-regulated for S0/S4 and R0/R4 comparisons or higher in susceptible or resistant for S0/R0 or S4/R4 comparisons). DEGs with a functional annotation related to immune response have black outlined symbols and gene names attached. Because there were many fin-TRIM genes clustered in QTL1, their name is given only once but their symbols are purple outlined

In our previous study, two novel quantitative trait loci (QTLs) were identified based on association of markers with CyHV-3 survival and gene lists within QTL boundaries were extracted [35]. Identifying DEGs within these QTLs can aid highlighting candidate genes affecting survival. Therefore, for these QTL DEGs, the level of expression change [Log_2_(fold-change)] was plotted against their genomic position in the QTL. As a reference, the significance [Log_10_(*p-* value)] of the test for association of markers with survival was also plotted against their genomic position (Fig. 5). From a few hundreds of genes located within the boundaries of each QTL1 and QTL2 intervals, 52 and 53 genes, respectively, were DEGs (Additional file 1: Table S1). For both QTLs, the distribution of DEGs along the QTL interval was more or less even. Also, no correlation appeared between the level of expression change and the significance of QTL marker association with survival. Therefore, neither the genomic position within the QTL nor the expression level could further help pointing out DEGs, which could be more important for CyHV-3 survival. However, what could help marking more promising candidate survival genes was that among these QTL DEGs, some were immune related genes. Inside QTL1 (Fig. 5a), a *ccl20* homolog (cypCar_00050111) was more highly expressed in susceptible than in resistant fish at day 4. Additionally, inside QTL1, a cluster of DEGs annotated as fish specific tripartite motif-containing (TRIM) genes (finTRIMs – ftr) are present. Inside QTL2 (Fig. 5b), a vitronectin b (*vtnb*) homolog (cypCar_00038793) was more highly expressed in resistant than in susceptible fish at day 0 and a Toll-like receptor-22 (*tlr22*) homolog (cypCar_00023948) was up-regulated at day4 in resistant fish. Outside these QTLs, the previously identified candidate gene for survival, Interleukin-10a (*il10a*) [33, 35], was more highly expressed in susceptible than in resistant fish at day 4 (Additional file 1: Table S1). The expression of Interleukin-10b (*il10b*), the paralog of *il10a*, was induced by the infection, but similarly in susceptible and resistant fish. Overall, combining data of QTL mapping with differential expression and functional annotation helped pointing out the more promising candidate CyHV-3 survival genes among all QTL genes.

### Comparing the temporal response to infection

**Fig. 6:**
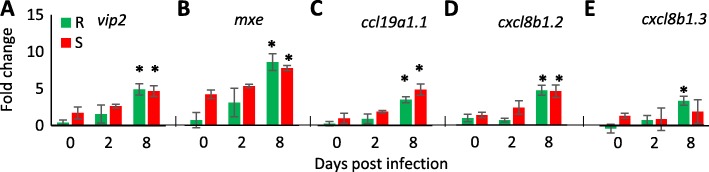
Relative expression of representative genes at further time points. Expression levels in susceptible (red bars) and resistant (green bars) fish at different days were measured using RT-qPCR, normalized to those of *ef1a* and the fold changes were calculated relative to the mean of one resistant family at day 0. Each panel refers to a different gene that was initially found to be differentially expressed between day 0 and 4 in susceptible or resistant fish in the RNA-Seq analysis. **a** and **b** interferon induced genes. Both *vip2* (**a**) and *mxe* (**b**) were up-regulated only in susceptible fish between day 0 and 4 based on the RNA-Seq data. **c** to **e** chemokine genes. Comparing the RNA-Seq data between days 0 and 4 identified *ccl19a1.1* (**c**) was up-regulated only in susceptible fish, while *cxcl8b1.2* (**d**) and *cxcl8b1.3* (**e**) were up-regulated only in resistant fish. None of the within-day comparisons between resistant and susceptible fish were statistically significant. In all panels, changes in expression that were significantly different from day 0 within the same fish type are marked by asterisks (*)

Since RNA-Seq was applied to susceptible and resistant fish only at day 4 post infection, it was interesting to study if susceptible and resistant fish elicited different responses or maybe similar responses but with different kinetics. Representative genes from the main identified immune pathways were chosen for RT-qPCR analysis in additional time points (day 2 and 8 post infection). Each of the selected genes was up-regulated in response to CyHV-3 infection in one of the fish types based on the RNA-Seq analysis. Representing the ‘response to virus’ (GO:0009615) GO term, which was the most enriched one in susceptible fish, two ISGs were chosen: cypCar_00024055 [Radical S-adenosyl methionine domain containing 2 (*rsad2*) also known as Viperin-2 (*vip2*)] and cypCar_00017679 [Myxovirus resistance E (*mxe*)]. Based on the RNA-Seq data, both genes were up-regulated DEGs at day 4 in susceptible, but not in resistant fish. In the RT-qPCR analysis, no induction was observed at day 2, whereas at day 8 the genes were equally induced in both susceptible and resistant fish (Fig. 6a and b). Thus, also in resistant fish ISGs are up-regulated in response to infection, but apparently at a later stage and not as part of the early induced immune response.

Three genes were chosen to represent the GO term ‘leukocyte migration’ (GO:0050900), which was the most enriched one in resistant fish and was also enriched in susceptible fish. One *ccl19a.*1 copy (cypCar_19885) to represent the main up-regulated CC chemokine in susceptible fish and two *cxcl8/il8* copies (cypCar_47570 and cypCar_45160) to represent the main up-regulated CXC chemokine in resistant fish. In the RNA-Seq analysis, cypCar_19885 (*ccl19a.*1) and cypCar_47570 (*cxcl8/il8*) were up-regulated DEGs at day 4 in susceptible, but not in resistant fish. In the RT-qPCR analysis, no induction was observed at day 2, whereas at day 8 the genes were equally induced in both susceptible and resistant fish (Fig. 6c and d). However, cypCar_45160 (*cxcl8/il8*) that was up-regulated at day 4 in resistant, but not in susceptible fish, was not induced at all at day 2, and at day 8 it was still induced in resistant but not in susceptible fish (Fig. 6e). Thus, analysis of representative chemokine genes at further timepoints indicated that genes, which were induced at day 4 in susceptible fish, were induced also in resistant fish but at a later infection stage. In contrast, the gene that was up-regulated at day 4 only in resistant fish, was not induced in susceptible fish at the selected time points. Taken together, the results suggested both qualitative and temporal differences between fish types in response to infection.

## Discussion

### Distinct transcriptional responses between susceptible and resistant fish

Previous studies have used transcriptomics to study carp host response to CyHV-3 infection using susceptible strains at different time points and different tissues [36, 37]. In the current study, for the first time for CyHV-3, the transcriptome of resistant fish was studied in comparison to that of susceptible fish, prior and after infection. Since the two fish types are considerably different, both genetically and phenotypically [18, 35], it was not surprising to find many transcriptional differences (840 DEGs) at day 0 (S0/R0) driven by basal genetic variation. As reported before, CyHV-3 infects both fish types, mortalities of susceptible fish start around day 8 post-infection and by day 6 post infection, viral loads in susceptible fish are significantly higher than in resistant fish [18, 38]. Thus, day 4 post-infection was chosen here to study the gene expression differences that can potentially explain why resistant fish more effectively control the viral load and survive. The response to infection of susceptible fish was distinctively different than that of resistant fish, with only 55 (9.3%) up-regulated DEGs shared between their responses (R4/R0 ∩ S4/S0). Furthermore, at day 4, the response of susceptible fish included 4.3 times more up-regulated DEGs than that of resistant fish, possibly indicating that the infection in spleen of susceptible fish had advanced further, eliciting a broader response compared to resistant fish.

Distinct transcriptional responses between susceptible and resistant fish of other species were described for other infections, such as during early infection stages (24 h) with Viral Hemorrhagic Septicemia virus (VHSV) in Rainbow trout (*Onchorhynchus mykiss*) [39] and late infection stages (during and after mortalities) of Infectious Salmon Anemia virus (ISAV) [40] in Atlantic salmon. Such studies, characterizing different disease stages, might not be comparable. Yet, when studies are comparable, some general features could be deduced. A recent study in Atlantic salmon reported that susceptible salmon fish at day 7 post infection with Infectious Pancreatic Necrosis virus (IPNV) had two times more up-regulated DEGs compared to resistant fish at the same time point, and less than 10% of these DEGs were shared [41]. Since the Atlantic salmon study and our carp study were done at a similar stage of infection when virus levels had elevated in susceptible fish, but mortalities had not yet started [18, 41], these similarities may suggest more general differences in transcriptional responses related to resistance.

Understanding resistance mechanisms beyond such general patterns requires further analyses at the level of enriched pathways and individual DEGs. Getting to this level of details in fish is challenging compared to mammals because many of the immune system gene families had expanded during fish evolution. Chemokines belong to one such expanded gene family. For instance, mammals have 24 different CC chemokine genes, while in ZF, thus far, 81 were identified [42]. The expansion of this gene family complicates the establishment of orthologuos relationships between fish and mammal chemokines that can help deducing on their function [43]. The recent carp-specific whole genome duplication [27] doubled the number of most chemokines compared to diploid bony-fishes including ZF (see the chemokine phylogenetic tree in Fig. 4a). This expansion in chemokine copy number had facilitated diversification in function of copies as reflected here by the transcriptional differences that correlated with resistance. For instance, among the three *ccl19* homologs found in ZF, one was more highly expressed (*ccl19a.1*) than the others in response to this infection. In this example, the two paralogs in carp of each ZF ortholog had similar expression levels. However, for *cxcl8/il8* diversification in expression levels in response to infection were observed not only between homologs found in ZF but also between carp paralogs of these orthologs. Previously, two lineages of *cxcl8/il8* were identified in carp and ZF, one (*cxcl8a*) was similar to human *CXCL8* gene and another (*cxcl8b* family) was unique to cyprinids [44]. The diversification levels we identified indicated that for studying mechanisms in carp based on transcriptional changes, the resolution of analysis should be down to copy specific expression and for gene families like chemokines, a phylogenetic reference is indispensable for interpretation of the results.

### Anti-viral immune response in susceptible fish

For susceptible fish, immune-related GO terms were most significantly enriched in DEGs and mainly the term ‘response to virus’, which includes interferons (IFNs) and interferon-stimulated genes (ISGs) [45]. Previous transcriptomic studies in susceptible CyHV-3 infected common carp, shared our observation of a wide response that is mainly immune related [36, 37]. More specifically, IFN-mediated response to CyHV-3 infection has been reported recently also for other carp strains with variable survival levels [38]. The IFN response in fish, similarly to mammals, acts as an important part of innate anti-viral immunity [46] and thus, induction of this pathway is not suprising. Interestingly, in contrast to susceptible fish, among the DEGs of resistant fish at day 4 post infection, IFNs and ISGs were absent. A few studies comparing between susceptible and resistant fish identified differences in their type-I IFN mediated anti-viral responses. Higher IFN response in susceptible fish was found also for Atlantic salmon at day 7 and 21 post infection with IPNV [41] and for late stage of ISAV infection [40]. These studies potentially suggested that an earlier activation of the IFN response could improve resistance. This has been suggested also by VHSV cell line infection model, where at 24 h post-infection the IFN response was significantly higher in cell cultures from resistant Rainbow trout fish compared to those from susceptible ones [39, 47]. However, our results show no indication of an earlier induction of IFN response in resistant fish. Expression of *mxe* and *vip2* carp homologs was not significantly elevated at an early infection stage (day 2) at either fish type. These genes in susceptible fish were induced at day 4 and were still induced at day 8 post infection while in resistant fish induction was observed only later (day 8). This delay in the IFN temporal response correlates with the delay in virus load elevation seen in resistant fish [18]. Thus, CyHV-3 resistance is not likely based on an earlier anti-viral IFN response, rather it seems that the IFN response is correlated to the elevation in viral loads during infection. This could have been the case also for these aformentioned studies. Similar correlation was found for Spring Viremia of Carp Virus (SVCV) in common carp as elevation of *ifna2* and *viperin* was correlated to the normalized viral copy numbers across six time points and four strains with variable resistance levels [38]. Overall, despite differences in species and viruses studied, our data uncovered differences in transcriptional response between susceptible and resistant fish, which together with similarities across fish species suggested these responses are potentially conserved and more general.

### Distinct chemokine profiles for susceptible and resistant fish

The most enriched GO term in the list of up-regulated genes in response to infection of resistant fish was `leukocyte migration’ that included mostly CC and CXC chemokines. Many more genes were up-regulated in susceptible fish than in resistant fish in response to CyHV-3 infection, including more chemokines. However, because of the fewer DEGs, the fold-enrichment of ‘leukocyte migration’ in resistant fish was higher than in susceptible, but most importantly included different genes. Chemokines act as chemo-attractants that promote leukocyte differentiation and migration. As such, one of their roles is to mediate between innate and adaptive immunity [43, 48]. Almost each chemokine type actually comprises a gene family with multiple copies in the ZF genome, let alone in that of the common carp. Hence chemokine genes exact annotation might not be perfect and their individual function far from being understood. Notably, subjected to the accuracy of the annotations from the ZF, the carp chemokines that were up-regulated in susceptible fish were different than those in resistant. Up-regulated in susceptible fish were predominantly chemokines that attract mono-nuclear leukocytes such as T-cells and macrophages, e.g., *ccl19* genes [49, 50] and *cxcl11* genes [51]. Contrary to susceptible fish, up-regulated in resistant fish were mainly the *cxcl8/il8* chemokines, which are known to attract neutrophils [44, 52]. Thus, according to chemokine profile induction, different arms of the cellular immunity are elicited by susceptible and resistant fish at this stage of infection.

As discussed earlier, CyHV-3 infected susceptible fish mount a clear IFN/ISG anti-viral response and the chemokine mediated attraction of mono-nuclear leukocytes fits with this response. In contrast, resistant fish showed a delayed IFN/ISG response and a chemokine profile compatible with attracting poly-nuclear neutrophils. Some of these chemokines were expressed specifically in resistant fish and others expressed in susceptible fish only at day 8 post infection when their mortalities start. At the later disease stage in susceptible fish, it is reasonable to associate induced neutrophil activity with the attempt to clear virus infected apoptotic cells. However, in resistant fish that contain lower viral loads in their tissues, and especially earlier during infection, it is less likely that neutrophils were simply recruited for the clearance of virus infected cells that become apoptotic early during infection [53]. Thus, this finding suggests that early activation of neutrophil is a part of the innate immune mechanism against the virus. The role of neutrophils in viral infections is largely unknown even in mammals, however some studies provided evidence for a possible beneficial role [54]. Mice infected with Herpes Simplex Virus [55] or with Neurotropic mouse Hepatitis Virus [56] that were depleted of neutrophils had elevated virus loads compared to controls, indicating that in some virus infections, neutrophils can play an important role in suppression of the virus. Thus, this neutrophil-mediated response may be part of an innate immune mechanism underlying resistance to CyHV-3 in carp.

## Conclusions

To enhance our understanding of viral resistance mechanisms, in this study we characterized the transcriptional response to CyHV-3 infection of susceptible and resistant fish. Susceptible and resistant fish differed in both the magnitude of response as well as in sets of regulated genes. A different immune-related response as seen here, may provide clues to why the outcome of the disease is different between susceptible and resistant fish. Expanding the analysis to further time points and possibly more tissues might strengthen our findings and yield a more comprehensive picture of the resistance mechanisms. However, in addition to the specific knowledge on carp CyHV-3 resistance, already now, some insights emerged that are shared with other species in response to other diseases regarding differences between susceptible and resistant fish. Therefore, this study contributes to the understanding of infectious disease resistance mechanisms and fish immunogenetics and to the development of sustainable and safe aquaculture.

## Materials and methods

### Sampling data

The production of carp families used in this study and how their resistance to CyHV-3 was measured, are described in [18]. In short, each family was assigned a phenotypic value of % survival based on at least two independent measurements in a cohabitation disease model. Three susceptible families (family % survival lower than 30%) were chosen from the genetic background of the cultured Yugoslavian food strain [57], and three resistant families (family % survival higher than 70%) from the genetic background of the feral strain Amur-Sassan [58]. The % survival of the selected families was determined beforehand (Fig. 1d in [18] describes the cumulative mortality curves of the six families used here). All families were produced and reared in our fish facility. To obtain samples for RNA-Seq, 20–50 naïve fish from each of the six families, with a mean weight of 30–50 g, were infected using our established cohabitation disease challenge model [18]. On day 0 pre-infection and day 2, 4 and 8 post-infection, spleen tissue from three euthanized fish from each family were quickly collected on ice and immediately stored in RNAlater. For spleen sampling, fish were euthanized by placing them in water with high concentration of anesthetics (2-phenoxyethanol), in which within a minute they become anesthetized and unconscious and after a couple more minutes die. Remaining unsampled fish were left in the cohabitation challenge to verify that the mortalities of families were as expected from the previous measurements done in [18]. The effectiveness of the challenge was monitored also by including susceptible koi fish as controls. Fish that were not sampled, either died from the disease or survived and recovered.

### RNA sequencing

RNA was extracted from spleen samples of three fish of each of the six families at time points 0 and 4 (a total of 36 samples) using RNeasy mini kit (Qiagen), according to the manufacturer’s instructions. RNA samples were sent on dry ice for RNA-Seq at Future Genomics Technologies (Leiden, NL), where RNA integrity and concentration were analyzed by Agilent Bioanalyzer 2100 total RNA Nano series II chip. All 36 individual samples passed the quality level and then 12 RNA pools were created, each containing equal amounts of total RNA from three fish of the same family and timepoint. The pooled RNA samples also passed the quality checks for quantity and integrity of the Bioanalyzer test. Barcoded sequencing libraries were prepared from a total of 12 pools (three replicates of each combination of susceptible/resistant family and day 0/4) and sequenced using Illumina HiSeq2500 flow cell to obtain 100 bp paired-end reads. After trimming of barcode and adaptor sequences, a total of 15–25 million clean paired reads were obtained from each pooled sample (Table 3). Raw sequence reads were deposited at the NCBI sequence read archive (SRA) under project number PRJNA565549 that includes 12 samples with the following accession numbers: SRR10120624, SRR10120623, SRR10120622, SRR10120621, SRR10120620, SRR10120619, SRR10120618, SRR10120617, SRR10120616, SRR10120615, SRR10120614 and SRR10120613.

**Table 3:** Raw RNA read pair counts for pooled samples

Pooled sample^a^	Number of clean raw read pairs	% mapping of reads^b^
R0_1	16,767,072	48.09
R0_2	22,925,459	21
R0_3	17,896,912	41.2
S0_1	18,800,570	35.94
S0_2	18,024,164	38.28
S0_3	18,505,908	42.97
R4_1	17,370,478	39.81
R4_2	19,861,892	41.95
R4_3	22,849,237	40.87
S4_1	15,788,290	36.16
S4_2	25,499,790	38.22
S4_3	19,324,230	39.91

^a^ *R* resistant, *S* susceptible, 0 – day 0 pre infection, 4- day 4 post infection

^b^ % mapping of reads to carp transcriptome from [30]

### Transcriptome analysis

Transcript quantification (the number of reads per gene) from the RNA-Seq data was performed using the Bowtie2 aligner [59] against the reference transcripts extracted from the NCBI (BioProject accession PRJNA73579, [30]). The Expectation-Maximization method (RSEM) was used for estimating maximum likelihood expression levels [60] via the PERL script align_and_estimate_abundance.pl with --est_method RSEM from Trinity protocol [61]. Differential expression analysis was done using the DESeq2 package in the R software [62]. Genes with at least two-folds difference in expression, an adjusted *P*-value for the difference of 0.05 or lower [63], and mean normalized read count (DESeq2 normalization) of above 30 in at least one treatment were considered DEGs. Four comparisons were analyzed to identify DEGs: between day 0 and day 4 in susceptible fish (S4/S0) and in resistant fish (R4/R0), as well as between susceptible and resistant fish at day 0 (S0/R0) and at day 4 (S4/R4). DEGs had higher or lower expression relative to the reference treatment, which is always in the denominator (e.g for S0/R0, R0 is the reference). Similarity between samples was evaluated by correlating the normalized expression values of all DEGs between each pair of the 12 samples and by hierarchical clustering of these correlation coefficients using centralized and log2 transformation [61] and R Bioconductor modules [64]. ‘Venny’ tool (http://bioinfogp.cnb. csic.es/tools/venny_old/venny.php) was used for creating the Venn diagram for all DEGs.

### Gene annotation and GO term analysis

Annotations for most genes were obtained from the published transcriptome of common carp [30]. In addition, the transcriptome sequences (BioProject accession PRJNA73579) were used as a query list for a homology search in the NCBI non-redundant (nr) protein database that was carried out with the DIAMOND program [65]. The search results were imported into Blast2GO version 4.0 [66] for gene ontology (GO) assignments. Integration of all annotation sources improved gene annotation of some genes.

Two online tools were used for GO terms enrichment analysis. In both cases, analysis was performed based on Fisher’s exact test with the significance threshold set to a *P*-value of 0.05 or less after correction for multiple testing using false discovery rate (FDR). GO term enrichment was determined by comparing the DEGs query list to the list from the background reference transcriptome for each GO term. One analysis was performed using PANTHER tool [67] and was based on carp gene annotations obtained from ZF (*D. rerio*) and therefore, using ENSEMBL stable IDs of ZF genes for both the carp DEGs list and the ZF reference database. A carp specific analysis was performed using AgriGO tool [68], using GO annotations obtained in this study by blast2GO. Since both tools gave similar significantly over-represented categories, PANTHER tool was used further for analyzing in details the enriched gene lists in each category and for reporting.

### Chemokine phylogenetic tree

A list of differentially expressed carp chemokines was extracted from the carp transcripts and supplemented with similarly annotated genes from the non-DEGs gene list to a total of 34 carp transcripts. Carp transcripts were annotated based mainly on the closely related ZF genes and therefore, gene names where used to extract the corresponding ZF gene transcript sequences from the Zebrafish Information Network (ZFIN.org). Sequences of these carp and ZF transcripts were BLAST searched against ENSEMBL cDNA database to find homologous gene transcripts in an outgroup species. Based on availability of homologous genes, the non-cyprinid species Red-bellied piranha (*Pygocentrus nattereri*) was selected as an outgroup for the phylogenetic analyses. All gene accessions and annotations used for the analysis are detailed in Additional file 1: Table S2. Transcript sequences from carp, ZF and red-bellied piranha, as they were found by the accessions listed in Additional file 1: Table S2, were aligned using ClustalW algorithm and the alignment was slightly improved manually. There were some differences in length and coverage between accessions, however, all these transcripts include a significant portion of the ORF of the genes ensuring correct gene alignments. A gene tree of chemokines was constructed based on this alignment, using the General Time Reversible model, number of differences distance, and the maximum likelihood tree construction method with 1000 bootstrap replicates. The tree with the strongest bootstrap support was chosen for representation. Both alignment and tree construction were done utilizing the Molecular Evolutionary Genetics Analysis (MEGA6) freeware [69].

### Expression levels analysis of chemokine gene copies

Carp chemokines were divided into clades based on their phylogeny. Carp gene copies were then numbered as copies of their phylogenetically related ZF gene. Normalized read counts from the RNA-Seq data were extracted for these carp genes and differences in gene expression were tested for chemokines in each clade using JMP14 software (SAS institute). Normalized read count data were Log_10_ transformed to equalize variances and transformed values were used as the analyzed variable in a one-way ANOVA followed by comparison among multiple means by Tukey-Kramer honestly significant difference test.

### Expression level of DEGs within survival QTLs

Two novel quantitative trait loci (QTLs) affecting CyHV-3 survival were previously identified by us [35]. Here, DEGs within these QTLs were identified. DEGs from all four pairwise comparisons were considered, since some of the differences in resistance might be due basic (day 0) differences in expression, while others due to expression differences only in response to the infection. For these QTL DEGs, absolute Log_2_(fold-change) values were plotted against their location (according to [35]) on the orthologous chromosome of the closely related ZF. At this time, carp genomic location is uncertain due to the discontinuous state of the carp genome assembly. Adapted from the previous data [35], markers in these QTL intervals were therefore mapped to their position in the ZF genome. The significance level Log_10_(*P*-value) of the association of markers with survival was plotted as a function of their ZF genomic position. DEGs were labeled based on the comparison in which they were identified.

### RT-qPCR analysis of representative DEGs

To study further the temporal response to infection, expression of genes representing the main differentially expressed pathways was studied at day 0 pre-infection and days 2 and 8 post-infection. RNA was extracted as described above from spleen samples of three fish per each of six families (three of each resistant and susceptible) from each of these three time points (total of 54 samples). cDNA was synthesized from RNA samples as described in [70]. Primers for selected immune response genes (Additional file 1: Table S3) were designed based on carp transcript accession sequence (cypCar) [30]. Primer pairs were verified to be copy-specific by their DNA melting curves. Based on results for common carp by our group [70], elongation factor 1-alpha (*ef1a*) was chosen as a reference gene. Calibration curves were generated for each gene to calculate amplification efficiency and the preferred amount of cDNA template to use. To generate a calibration curve, a pool of cDNA samples was created and serially diluted eight times, each time by a factor of four. Eventually, each cDNA sample was diluted 1:4 with ddw prior and 2 μL of cDNA template were added to wells containing 18 μL of reaction mastermix [6 μL water, 2 μL of primer mix (forward + reverse, 2 μM) and 10 μL Platinum SYBR Green qPCR SuperMix-UDG (Invitrogen)]. The thermal cycling profile was performed on LightCycler® 96 machine (Roche) consisted of pre-amplification hot start segment (95 °C for 2 min, 50 °C for 2 min), amplification segment (95 °C for 15 s, 60 °C for 30 s, 72 °C for 15 s, with a single fluorescence measurement), repeated for 40 cycles and a dissociation curve segment (95 °C for 10 s, 65 °C for 60 s and ramp up to 97 °C, with increment of 0.2 °C per second and continuous fluorescence measurement). Threshold cycle (Ct) values were determined by LightCycler® 96 (Roche) designated software at fluorescence levels within the exponential phase of amplification and above the background noise level for each gene (0.25–0.45 range).

Ct for each sample was measured in technical duplicates. When two duplicates varied significantly, they were repeated on a new plate with a shared common sample that was added to every plate for normalization between plates. The raw Ct data for each sample is given in Additional file 1: Table S4. For each sample, relative expression calculation was based on the mean of these technical duplicates. Relative expression of target genes was calculated using the “−ΔΔCp with efficiency correction” calculation method [71]. Technical means of three individuals belonging to the same families were averaged for the reference gene (*ef1a*) and for the target gene. Expression levels of target genes were then calculated relative to the mean value of the reference gene and to the mean value of one resistant family at day 0. For statistical comparisons, the relative expression values were transformed [Log_2_(fold-change)] to obtain normally distributed values and equal variances and the comparisons among days and fish types were done on the average values of three families. Differences between days 2 and 8 post-infection and day 0 pre-infection in each phenotypic group were evaluated by Dunnett’s test and between each phenotype within day by Student’s T-test. Statistical analyses for all parts of the results were done using the JMP14 software (SAS institute, NC).

## Supplementary information

**Additional file 1: Table S1.** Differentially expressed genes within previously identified CyHV-3 survival QTLs. **Table S2.** Accessions used for the chemokine phylogenetic tree. **Table S3.** Gene cypCar IDs and primers used for qRT-PCR. **Table S4.** Raw cycle threshold (Ct) data for individual fish in all treatments.

## Data Availability

The datasets supporting the conclusions of this article are included within the article (and its additional files). Raw sequence reads from the RNA-Seq analysis were deposited at the NCBI sequence read archive (SRA) under accession project number PRJNA565549 (https://www.ncbi.nlm.nih.gov/bioproject) .

## Abbreviations

ANOVA: Analysis of variance
Blast2GO: A method for annotating genes based on their sequence similarity
CC chemokine: Type of chemokines defined by their adjacent cysteine (CC) protein motif
CXC chemokine: Type of chemokines defined by cysteine-other-cysteine (CXC) protein motif
CyHV-3: Cyprinid herpes virus 3
cypCar: Unique ID for a gene in the common carp genome
DEG: Differentially expressed gene
dsDNA: Double stranded DNA
GO: Gene ontology
IFN: Interferon
IL: Interleukin
IPNV: Infectious pancreatic necrosis virus
ISAV: Infectious salmon anemia virus
ISG: Interferon stimulated gene
KHV: Koi herpes virus
NCBI: National center for biotechnology information
QTL: Quantitative trait locus
R: Resistant
RNA-Seq: RNA sequencing
RSEM: Expectation-maximization method
RT-qPCR: Real-time quantitative PCR
S: Susceptible
SRA: Sequence read archive
SVCV: Spring viremia carp virus
TLR: Toll-like receptor
VHSV: Viral hemorrhagic septicemia virus
ZF: Zebrafish
